# Supplementary material for: BlotIt—Optimal alignment of Western blot and qPCR experiments
Source: PLoS One. 2022 Aug 10;17(8):e0264295. doi: 10.1371/journal.pone.0264295 (PMC9365137; doi:10.1371/journal.pone.0264295)
Supplement: S1 Raw images — (PDF) [file pone.0264295.s001.pdf]

In this work no self-produced western blot or gel data was used. All data in our manuscript is either simulated or taken from the already published work of Kok et al.
